# Prostaglandin E_2 _binding peptide screened by phage displaying: a new therapeutic strategy in rheumatoid arthritis

**DOI:** 10.1186/1476-511X-10-75

**Published:** 2011-05-14

**Authors:** Dongmei Yan, Weiwei Han, Qinzhu Bai, Xiangfeng Zhao, Xiao Han, Bairong Du, Xun Zhu

**Affiliations:** 1 Department of Immunology, Norman Bethune College of Medicine, Jilin University, Changchun, China; 2 Key Laboratory for Molecular Enzymology and Engineering, the Ministry of Education, Jilin University, Changchun, China

## Abstract

**Objective:**

To investigate the therapeutic potential and mechanism of action of the mimotope of PGE_2 _receptor EP4 (PBP, named by our team) screened by phage displaying technique in the treatment of adjuvant-induced arthritis (AA).

**Methods:**

Freund's complete adjuvant-induced arthritis was induced in Wistar rats. At the first clinical sign of disease, mice were given with daily injections of PBP or saline for 21 days. Disease progression was monitored by measurement of paw swelling. Inflammation and joint destruction were assessed histologically. The IL-1β and TNF-α were studied by ELISA in the ankle steeps of arthritis model. The degree of proliferation and apoptosis of synoviocytes of RA patients were assessed by CCK-8 kit and AnnexinⅤ-FITC/PI respectively.

**Results:**

PBP-treated animals displayed significantly less cartilage and bone destruction than model controls. Tumor necrosis factor α and IL-1β expression were reduced after PBP treatment. The proliferation and apoptosis of synoviocytes of RA patients were influenced by PBP.

**Conclusions:**

The data support the view that PBP is a potential therapy for RA that may help to diminish both joint inflammation and destruction. And the activities of PBP are related with the effect on synoviocytes directly.

## Introduction

RA is characterized by systemic and local inflammation resulting in cartilage and bone destruction. Non-steroidal anti-inflammatory drugs (NSAIDs), which represent an effective therapy for treating RA, elicit their effects by inhibiting cyclooxygenase (COX) activity and blocking the downstream production of prostaglandin, including prostaglandin E2 (PGE_2_). And the major medicine treating RA is COX-2 selective inhibitor. However, this kind of medicine has side-effects such as cardiovascular effects [1,2], which have limited its use. PGE_2 _is the most important molecule in the pathogenesis of rheumatoid arthritis [3], which can be secreted by a lot of cells including macrophage cells and synovial fibroblast. Moreover, PGE_2 _is one of the main products of cyclooxygenase in a number of physiological settings. The diverse effects of PGE_2 _may be accounted for in part by the existence of four receptors, designated EP1, EP2, EP3, and EP4 [4,5], and heterogeneity in the coupling of these receptors to intracellular signal transduction pathways. In RA, EP2 and EP4 receptors, especially EP4 receptor [6], are the major ones which couple to the Gs-type G protein leading to stimulation of cAMP.

Then specific mimotope of PGE_2 _receptor may prevent the binding between PGE_2 _and receptor, which may be an effective treatment method on RA. Phage displaying technology has abroad application in the study of reorganization of protein molecules, development of new vaccine and new drug. Its greatest advantage is providing some libraries where the target-specific binders can be selected conveniently. So we have used the phage displaying technique to select C-X7-C peptides with PGE_2 _as the target and named these peptides PBPs (PGE_2 _binding peptide) and regarded the PBPs as a mimotope of EP4 [7]. Moreover, some reports have demonstrated that the key binding sites between PGE_2 _and receptor are related with arginine and threonine [8,9]. So we have selected one peptide of which the sequence is CANRTSKNC to synthesize.

In the present study, we generated RA rat models and treated them with PBP. It was found that this treatment could reduce arthritis severity and footpad swelling. To investigate the therapeutic effect and the mechanism of this mimotope in RA, we detected its anti-inflammatory effect furthermore and demonstrated this mechanism *in vivo *and *in vitro*.

## Materials and methods

### Animals

Male Wistar rats weighing 160 g-180 g, were purchased from the animal center of our university. All animals were placed at a controlled temperature (22°C-28°C), and a regular light/dark cycle (6:00 to 19:00 h, light), and all animals had free access to food and water.

### Chemicals

PGE_2 _was bought from Jingmei Lt and PGE_2 _ELISA kit was from the Suzhou Hematology Research Centre. ELISA kits for IL-1β and TNF-α were Ebioscience products. Celecoxib was purchased from Pfizer Pharmaceuticals Limited product. All enzymes were from TaKaRa Biotechnology (Dalian) co.ltd. Freund's complete adjuvant (FCA) was from Beijing Dingguo Biotechnology co. ltd. CCK-8 kit was purchased from Dojindo.

### Molecular modeling

Theoretical structure model of PBP and PGE_2 _were obtained using the HOMOLOGY module of the Insight II 2000 software firstly. The surface and interior between PBP and PGE_2 _were distinguished and the best connection between them was determined with computer graphics technology. The most acceptable solution was determined and optimized using Discover Module of Insight II (2000) software package. The Discover_3 program was used to generate the low-energy conformation of the complex.

### Induction and treatment of arthritis in rats

Rat AA was induced as previously described [10]. Briefly, The rats were immunized by subcutaneous injection into the left-hind paw with 0.1 ml Freund's complete adjuvant (FCA), containing 10 mg heat-inactive BCG in 1 ml paraffin oil. All procedures were approved by University Committee on the use and care of laboratory animals. Arthritis usually developed 6 days later after immunization. The rats were considered to have arthritis when significant changes of redness and/or swelling were noted in the digits or other parts of the paw. The animals were assigned to one of five groups at random, in which they were treated intraperitoneally with 10 ug/kg, 20 ug/kg, and 40 ug/kg PBP per injection, with 12.5 mg/kg celecoxib 0.5 ml as positive control, with 0.5 ml saline as negative control. Treatment was started on the seventh day after this disease was induced. All groups were treated daily for 21 days. Thereafter, the rats were killed by cervical dislocation and the hind paws and sera were collected and used for further analysis. The *in vivo *experiments were performed with 6 rats per group.

### Evaluation of arthritis activity

Rats were inspected daily for signs of arthritis by an independent observer who was not aware of the treatment. After 21 days of treatment, rats were killed and their paws were processed for histopathological evaluation.

### ELISA for inflammatory cytokines IL-1β and TNF-α in ankle steeps

In order to detect the cytokine level in the ankles, whole ankle joints were cut after rats were anaesthetized and dipped in PBS for 24 h after the skin and parenchyma were cut open. The ankle steeps were collected and stored in a -20°C refrigeratory for the next tests. TNF-α and IL-1β in the samples were measured using enzyme-linked immunoassay kit (ebioscience company rats TNF- a and IL-1β ELISA kit), according to the manufacturer's protocol. Briefly, ankle steeps were added to wells from 96-well plate precoated with 100 ul/well capture antibody and incubated for 2 h at 37°C. After wash, detection antibody were added and incubated for 1 h at 37°C. After another wash, horseradish peroxidase (HRP) labeled anti rat IgG goat antibodies were added and also incubated for 2 h at 37°C, After the final wash, the presence of HRP-labeled Abs was revealed by addition of o-phenylenediamine (OPD) and the reaction was stopped with sulfuric acid (2N). The coloration was evaluated by optical density at 490-630 nm.

### Histology

Whole ankle joints were fixed in 5% formalin at 4°C for 24 h. Subsequently, joints were decalcified in 10% ethylene diamine tetraacetate (EDTA) in buffered formalin (5.5%) for 4 weeks, and processed for histology by routine paraffin embedding, sectioning (4 μm), and staining using hematoxylin and eosin (H&E) and examined for the degree of synovitis and bone erosions by microscopic evaluation in a blinded manner as described earlier. Bone erosions were scored using a semiquantitative scoring system from 0 to 4 (0 = no erosions, 4 = extended erosions and destruction of bone).

### Isolation of synoviocytes

Synovial tissues were obtained from RA patients who met the American Rheumatism Association criteria for classification and had undergone joint replacement surgery (5 women, 2 men; 55 ± 2 years). The study was approved by the local ethics committee, and informed consent was obtained from each patient enrolled. The synovial samples were digested and subsequently cultured as described by Zimmermann et al [11]. Synovial fibroblasts were isolated by enzymatic digestion of synovial tissues obtained. Synovial tissues were minced into 3-5 mm pieces and treated for 4 h with 4 mg/ml of typeⅡcollagenase (Sigma Chemical Co., St Louis, MO, USA) in Dulbecco's modified Eagle's medium (DMEM; Gibco BRL., Grand Island, NY, USA) at 37°C in 5% CO_2_. Dissociated cells were then centrifuged at 2000 rpm and resuspended in DMEM supplemented with 20% fetal calf serum (FCS), 100 units/ml penicillin, and 100 mg/ml streptomycin, and plated in 6 well culture plates. The next day, cultured plat was vigorously rinsed to remove non-adherent cells with fresh DMEM, and adherent synovial fibroblasts were cultured in DMEM supplemented with 10% FCS. The cultures were kept at 37°C in 5% CO_2_, and the medium was replaced every 4 days. The cells were morphologically homogeneous and had the appearance of fibroblast, with typical bipolar shown. We named them as HSY (Human Synoviocyte Yan). All the experiments described below were conducted using cells at the 4-10 passage. The experiments were done as follows.

### Synoviocytes proliferation assay

The synoviocytes were suspended in DMEM medium with 10% FCS at a concentration of 1 × 10^5 ^cell/ml. The cell suspension of 100 μl were added to 96-well flat-bottomed culture plate and incubated at 37°C, in 5% CO_2 _for 24 h. After the cells adhered, PBP with different concentrations were added to the well, 5 μM, 2.5 μM, 1.25 μM, 0.625 μM, 0.3125 μM, and DMEM with 10% FCS as a negative control and incubated at 37°C, in 5% CO_2 _for 48 h. Then incubation were added with 10 μl WST-8 from CCK-8 kit (Dodindo, Japan) to each well, oscillated for 1 min on oscillator and incubated at 37°C and in 5% CO_2 _for 1.5 h continuously. After incubation, the absorbance (A) was measured at 450 nm.

### Apoptosis detection kit (Annexin V binding)

The assay was performed according to the instructions of the manufacturer. FLS were seeded at 1 × 10^5^cells/well and incubated for 24 h, then were treated with 5 μM PBP and 40 μM celecoxib, and PBS (phosphate-buffered saline) as a negative control. After 24 h, Cells were trypsinized, counted and sedimented, then washed with cold PBS, and resuspended in 500 μl binding buffer. Fluorescein isothiocyanate conjugated Annexin V (10 μl) and PI (10 μl) were added to each samples, and the mixture was incubated at 4°C in the dark for 5 min. Then the cells were immediately subjected to FACS analysis (BD FAC-S Calibur, USA).

### Hochest 33342 and PI staining

Cells growing in exponential phase were seeded at 2.5 × 10^4 ^cells in 2 ml of DMEM with 10% FCS in a 6 well culture plate with a cover slip and incubated in a 5%CO_2 _atmosphere. Cells were exposed to 5 μM PBP and 40 μM celecoxib, and PBS as a negative control. The morphology of treated and untreated cells was examined following incubation for 72 h. Cells were washed with PBS three times and then stained with 10 μl Hoechst33342 nuclear dye (working concentration was 5 μg/ml) in 2 ml of DMEM for 10 min at 37°C. After washing with PBS 3 times, cells were added with 2 ml new of DMEM and 30 μl PI (working concentration was 1.5 μg/ml) for 10 min at 4°C. After another washing with PBS for 3 times, images were obtained by confocal fluorescence microscope (Olympus-FV1000, Japan). The average of the apoptotic cells in each of the respective groups was determined as a percentage of the control (untreated cells).

### Statistical analysis

Differences between treatment groups were tested by one-way ANOVA and unpaired two-tailed Student' test. The data were expressed as the mean ± SEM. Analysis of variance of repeated data measured with SPSS 13.0, and P values less than 0.05 at 95% confidence level were considered significant.

## Results

### 3-D modeling structures of PBP and PGE_2_

**Figure 1:**
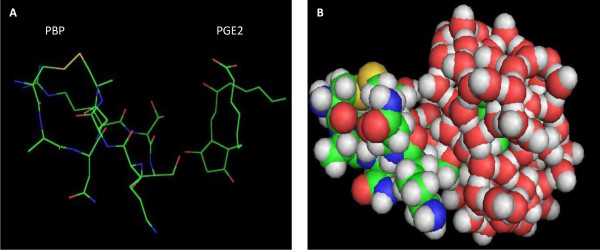
**The optimized complex structure of PBP and PGE**_**2 **_**using molecular docking and molecular dynamics methods**. Stick and sphere model were showed in A and B respectively.

The 3-D complex structure suggested that there formed hydrophobic interaction between PBP and PGE_2 _(Figure 1A and 1B), and the interaction energy between PBP and PGE_2 _was -11.4036 KJ/MOL, in which the VDH is -5.38675 KJ/MOL. So certain conclusions and predictions could be drawn from the results that the interaction between PBP and PGE_2 _had a relatively stable structure.

### PBP reduces the paws inflammation of AA

**Figure 2:**
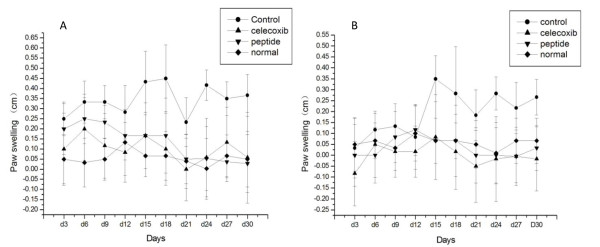
**Effects of the PBP on suppression of joint and tissue swelling in AA**. The paw swellings of rats were evaluated by an inelastic tape measure. All groups treated with PBP showed a 70% decrease in paw swelling compared to model controls, P < 0.05. And peptide treatment groups showed a marked destruction and infiltration compared with model groups. (A: the primary swelling of ankle joint. B: the secondary swelling of ankle joint).

**Figure 3:**
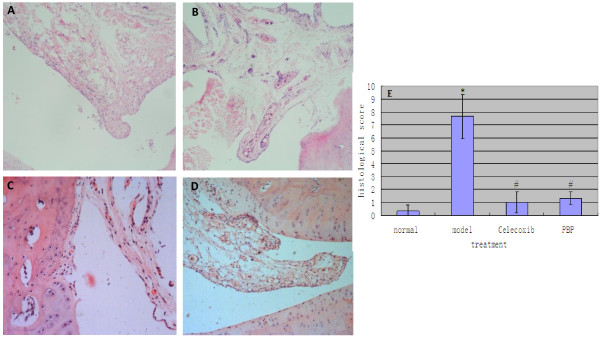
**Changes of the histological morphology of rats ankle joints by H&E staining**. The effect of PBP treatment on synovial inflammation was also assessed by histology. Wild-type rats showed no inflammation and destruction at all by H&E (A) from histopathology of the ankle joints, while model groups showed heavy cell infiltration, neovessel growth, synovial cell proliferation, and pannus formation in tissues of AA rats (B). Peptide treatment groups showed a less marked destruction and infiltration compared with model groups (C). Celecoxib treatment groups showed the same change as peptide treatment (D). (E): Effect of PBP on the clinical and pathological manifestations of rat adjuvant-induced arthritis. Note: 0: normal; 1: the extent of inflammatory cells' infiltration to the joint tissues; 2: synovial lining cell hyperplasia; 3: pannus formation; 4: joint cartilage layers destruction. *; P < 0.05 compared with normal. #; P < 0.05 compared with model.

To examine whether PBP therapy could be effective in the treatment of AA, rats were given with a daily intraperitoneal injection of various dosages of PBP or saline for 21 days. All animals were treated on the seventh day after immunization. All groups treated with PBP showed a 70% decrease in paw swelling compared to controls (P < 0.05) (Figure 2). There was no clear dose dependency in peptide treatment groups, suggesting that all dosages were in the therapeutic range with regard to effects on clinical signs of arthritis. The effect of PBP treatment on synovial inflammation was also assessed in histology. Wild-type rats showed no inflammation and destruction at all by H&E (Figure 3A) from histopathology of the ankle joints, while model groups showed heavy cell infiltration, neovessel growth, synovial cell proliferation, and pannus formation in tissues of AA rats (Figure 3B). And peptide treatment groups showed a marked destruction and infiltration compared with model groups (Figure 3C). It was found the histologic score decreased after PBP treatment compared with model groups (P < 0.05) but did not reach the normal level (Figure 3E).

### PBP pervasively suppresses TNF-α and IL-1β in inflammatory tissues of AA

**Figure 4:**
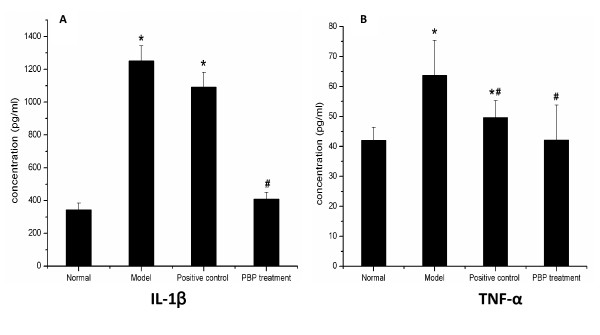
**Concentration of IL-1β and TNF-α in joint. ( ± s, n = 6)**. ELISA showed that the level of IL-1β and TNF-α in the ankle steep were reduced by intraperitoneal PBP. *; P < 0.05 compared with normal. #; P < 0.05 compared with model.

In particular, cytokines secreted from synoviocytes, such as TNF-α and IL-1, were considered major determinants in the perpetuation of arthritis [12]. TNF-α is a pro-inflammatory cytokine which may induce the proliferation of synoviocytes and enhance their production of cytokines such as TNF and IL-1 in situ, which largely show the pathology of arthritis. It also largely accounts for the pathology of autoimmune-related arthritis [13]. We then studied whether the level of TNF-α and IL-1β was decreased in the synovium of AA by PBP. ELISA showed that the level of TNF-α and IL-1β in the ankle steep were reduced by intraperitoneal PBP (Figure 4). There was also no obvious dose dependency in peptide treatment groups (data not shown).

### Effects and Mechanism of PBP Inhibitors on Synovial

**Figure 5:**
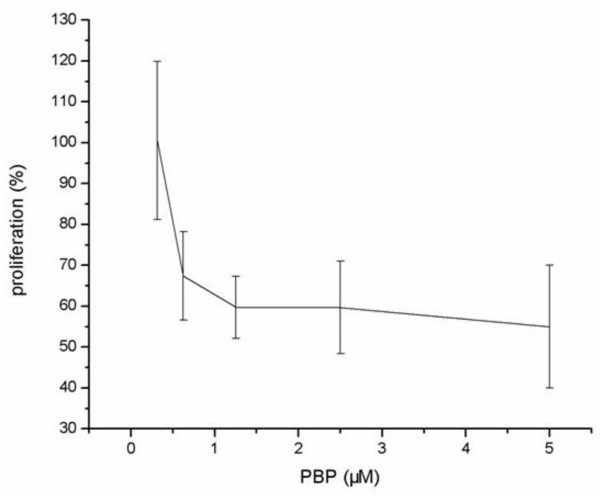
**Effects of PBP on synoviocytes viability**. Synoviocyte viability was assessed by CCK-8 assay, with control cells taken as 100%. Synoviocytes were incubated for 24 h with PBP (0.3125 μM, 0.625 μM, 1.25 μM, 2.5 μM, 5 μM).

Synovial hyperplasia was thought to play an important role in the development of AA which was caused by an increased rate of proliferation of FLS. Through CCK-8 assays, we had observed that PBP treatment groups could inhibit the synovial fibroblast proliferation compared with non-PBP groups. The inhibitory percent could arrive at about 50% (Figure 5). This was the evidence that PGE_2 _might be a mitogen which could promote synovial fibroblast proliferation. Therefore, this peptide through screening phage library with PGE_2 _as a target could inhibit PGE_2 _to interact with its receptor EP4 [6].

**Figure 6:**
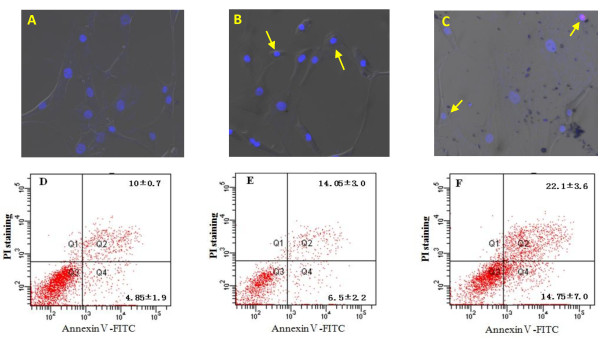
**Synoviocytes apoptosis induced by PBP**. **A-C: Apoptosis detected using cofocol fluorescence microscope**. Synoviocytes were incubated for 24 h with 5 μM PBP (B) and 40 μM celecoxib (C), and PBS as a negative control (A). They were stained by Hoechst33342 and PI. We had observed that viable cells, apoptotic, and necrotic cells. On the one hand, the Hoechst33342 dye stained the nuclei of all cells; therefore apoptosis might show nuclear changes such as chromatin condensation and nuclear fragmentation. So in PBP treatment groups, we could see these changes (see what was directed by arrow in B and C). On the other hand, PI uptake indicated the loss of membrane integrity characteristic of necrotic and late apoptotic cells. **D-F: Apoptosis detected using FACS**. Synoviocytes were incubated for 24 h with 5 μM PBP (E) and 40 μM celecoxib (F), and PBS as a negative control (D). They were stained by AnnexinⅤ-FITC and PI. Then, the viability was determined using the FCM. AnnexinⅤ-FITC (-) and PI (-), living cells; AnnexinⅤ-FITC (+) and PI (-), early apoptotic cells; AnnexinⅤ-FITC (+) and PI (+), late apoptotic cells and necrotic cells. The numbers showed the percentages of each fraction.

On the other hand, two methods were used to value the effects of PBP on FLS apoptosis. From the pictures of laser scanning confocal fluorescence microscope (Figure 6A-6C), we had observed viable cells, apoptotic, and necrotic cells. Moreover, the Hoechst33342 dye stained the nuclei of all cells; therefore apoptosis might show nuclear changes such as chromatin condensation and nuclear fragmentation. So in PBP treatment groups, we could see these changes (see what was directed by arrow in Figure 6B). While PI uptake indicated the loss of membrane integrity characteristic of necrotic and late apoptotic cells because PI was excluded from viable and early apoptotic cells (Figure 6C); in combination with fluorescence microscopy, selective uptake of the two dyes allowed one to monitor the induction of apoptosis in intact cultures and to distinguish it from non-apoptotic cell death (necrosis). Necrosis was characterized in this system by nuclear PI uptake without chromatin condensation or nuclear fragmentation, but this did not reach statistical significance. We also used AnnexinV-FITC and PI double staining with FACS to detect the effect of PBP on FLS apoptosis. We could see the percent of early apoptosis cells was 6.5 ± 2.2% in PBP treatment groups which increased by 1.7% than FLS negative control groups. While the percent of later apoptosis and necrosis was 14.05 ± 3.0, which exceeded FLS negative control groups by 4.05% (Figure 6D and 6E).

## Discussion

In 2002, McCoy and their groups showed that prostaglandin EP4 receptor, but not EP1, EP2, EP3 receptor-deficient mice decreased the incidence and severity of disease in the mouse arthritis model, suggesting that the mimotope of the prostaglandin EP4 receptor might provide novel agents for the treatment of rheumatoid arthritis [2]. Moreover several researchers had found that mimotope of the prostaglandin EP4 receptor such as CJ-042,794 [6,14] had the comparable effects on inhibiting inflammation process compared with non-selective COX inhibitor (piroxicam) and selective COX-2 inhibitor (rofecoxib). So how to select the mimotope of the prostaglandin EP4 receptor became the key point for searching effective medicines [15,16].

Through phage-peptide library, high affinity and specific peptides can be isolated which could be made into a new type medicine of treating diseases [17,18]. Phage displaying technology is a high-throughout screening (HTS) assay for studying the binding site of ligands and receptors, searching for the ligand molecules with high affinity and detecting the dimensional structure epitopes of unknown protein, which has an abroad application in the study of reorganization of protein molecules, development of new vaccine and new drug. Moreover, it plays an important role on the simulating epitopes, identifying the major animos between proteins interaction. The Ph.D-C7C is to link the genotype of short peptide displaying on the phage surface with the phenotype closely. Rapid enrichment of library clones encoding binding polypeptides is achieved by phage library incubation with a target followed by removal of the non-reacting phage and amplification of binder clones in the host bacteria [19-21]. Usually, three to five rounds of panning are sufficient to enrich for binding peptide sequences. In our studies, we have selected the mimotope of the prostaglandin EP4 receptor, which is named by PBPs. Moreover, we had employed the Molecular Docking technology with Insight II 2000 software in SGI working station to analyze the three-dimensional structure of PBP and PGE_2 _[22]. The lower energy indicates the high affinity between these two molecules, which provides the theoretical foundation for the next research.

With respect to the potential as a drug target in treating RA, our findings indicate that selective inhibition of PGE_2 _EP4 receptor will decrease the inflammation in ankles. This should markedly shift the balance away from the bone resorption that mediates osteolysis. It is of interest to see if future studies designed to investigate the effects of Cox-2 inhibition on the various other factors that stimulate inflammation and bone resorption in fibroblasts, as well as clinical trials, support this paradigm.

Although several labs have been able to demonstrate that Cox inhibitors can effectively block PGE_2 _synthesis, it has been demonstrated that this treatment can inhibit pro-inflammatory cytokine synthesis (e.g. TNF-α and IL-1) by these cells [23,24]. Thus, it remains unclear how NSAID effectively inhibit synoviocytes proliferation. Synoviocytes were the ultimate targeting cells of pathological change of arthritis [25]. PGE_2 _is an autocrine factor in rheumatoid arthritis, so its effect on the proliferation and apoptosis of synoviocytes should not be neglected. Through analysis of our results, it can be found that the prevention of interaction between PGE_2 _and its receptor EP4 by PBP can affect the cells proliferation in some degree, which is extremely beneficial for the treatment of RA.

Whereas IL-1 and TNF might be more important in the processes leading to cartilage and bone destruction and in limiting mechanisms involved in cartilage repair [26-28]. Tumor necrosis factor (TNF) antagonists (adalimumab, etanercept, infliximab) and TNF inhibitors (certolizumab pegol, golimumab) have been approved under the development respectively [29,30]. Interleukin 1 (IL-1) antagonist (anakinra) has been approved to treat RA [31]. So these two cytokines are better value to assess the degree of the arthritis and also good targets for treating the disease. It can be also found that PBP contributed to the reduction of pro-inflammatory cytokines TNF-α and IL-1, which maybe another mechanism of its anti-arthritis effects.

In conclusion, our results provide new relevant data of the mimotope activ**i**ties of PGE_2 _receptor. PBP as a mimotope of EP4 has anti-inflammatory activities, which has been proved *in vivo *and *in vitro*. So the peptide screened by phage displaying technique has relevant therapeutic potential to the treatment of arthritis diseases.

## Competing interests

The authors declare that they have no competing interests.

## Authors' contributions

XZ and DMY designed the study, WWH performed the bioinformation sequence and structural analysis, and others performed the experiments. DMY drafted the manuscript. All authors read and approved the final version of the manuscript.
